# Investigating the Correlation Between Choroidal Alteration and Visual Function Metrics in Dysthyroid Optic Neuropathy

**DOI:** 10.1167/tvst.15.6.38

**Published:** 2026-06-29

**Authors:** Tengbo Rao, Yi Wang, Xuemin Li, Jichao Zhou, Debo You, Wencan Wu, Lingge Suo, Jiarui Yang

**Affiliations:** 1 Department of Ophthalmology, Peking University Third Hospital, Beijing, People's Republic of China; 2 Beijing Key Laboratory of Restoration of Damaged Ocular Nerve, Peking University Third Hospital, Beijing, People's Republic of China; 3 State Key Laboratory of Ophthalmology, Optometry and Vision Science, Eye Hospital, Wenzhou Medical University, Wenzhou, People's Republic of China

**Keywords:** dysthyroid optic neuropathy (DON), choroidal alteration, wide-field optical coherence tomography angiography (OCTA), visual-evoked potential (VEP), visual field test

## Abstract

**Purpose:**

This study evaluates the relationship between choroidal and optic disc alterations and visual function metrics (visual acuity, pattern visual evoked potential [P-VEP], and visual field) in patients with dysthyroid optic neuropathy (DON).

**Methods:**

A retrospective cross-sectional study included 38 patients with thyroid eye disease (TED). Three-dimensional choroidal data, including thickness, vascular metrics, and optic disc measurements, were obtained using TowardPi wide-field optical coherence tomography angiography (OCTA). Visual function was assessed using P-VEP and automated perimetry.

**Results:**

The study analyzed 27 eyes with inactive TED, 22 with active non-DON TED, and 27 with DON. Visual acuity worsened with TED severity (*P* = 0.024), and mean sensitivity (MS) decreased (*P* = 0.024). P-VEP amplitude at 0.25 degrees was lower in DON (*P* = 0.024). Significant correlations were found between optic disc choroidal vascularity index and MS (*R* = 0.413, *P* < 0.001), nasal superior region choroidal metrics and MS (*R* = 0.450, *P* < 0.001), and retinal nerve fiber layer blood flow density with MS (*R* = 0.387, *P* = 0.001) and P-VEP amplitude at 0.25 degrees (*R* = 0.311, *P* = 0.021).

**Conclusions:**

Choroidal alterations are closely linked to visual function in DON. Choroidal thickness (CT) and vascularity may serve as biomarkers for assessing disease activity and visual impairment, aiding clinical management.

**Translational Relevance:**

This study identifies choroidal alterations as potential biomarkers for assessing disease activity and visual impairment in DON, which can aid in clinical decision making and personalized patient management.

## Introduction

Thyroid eye disease (TED) is an autoimmune disorder that primarily affects the orbit, leading to inflammation and swelling of the extraocular muscles, along with an increase in orbital connective and adipose tissue.^1^ Dysthyroid optic neuropathy (DON), the most common vision-threatening condition in advanced TED, is characterized by decreased visual acuity, constricted visual fields, and changes in visual-evoked potentials (VEPs), including alterations in amplitude and latency.^2^^,^^3^ However, no standardized criteria for DON diagnosis currently exist, and visual changes often manifest late in the disease course, making early-stage diagnosis particularly challenging.^4^ Additionally, functional tests such as VEP are subjective and can be influenced by ophthalmic and neurological conditions, reducing the reliability of clinical findings and causing variability in assessing disease severity.^5^ Therefore, there is an urgent need for objective biomarkers to evaluate disease activity and severity, especially in the early stages.

The choroid, major vascular layer of the posterior eye, is essential for delivering oxygen and nutrients to the outer retinal layers. In TED, mechanical compression caused by enlarged extraocular muscles and orbital adipose tissue has been reported to reduce blood flow in the short posterior ciliary arteries, which supply the choroid.^6^^,^^7^ This suggests that the choroid could serve as a valuable indicator of TED progression. In our previous studies, we also found that with the progression of TED stages, there is a decrease in choroidal blood flow, proliferation of the stroma, and changes in both the structural and blood flow parameters of the optic disc.^8^ The link between structural changes and visual function remains underexamined, calling for a comprehensive study.

This research investigates the association between visual function changes and wide-field choroidal and optic disc alterations across TED severities, particularly DON. It aims to identify the visual function metric most strongly correlated with structural changes, offering insights for improved clinical management.

## Materials and Methods

Thirty-eight patients with TED received comprehensive ocular examination, including best-corrected visual acuity (BCVA), refraction, intraocular pressure (IOP) measurement, indirect ophthalmoscopy, Hertel measurements, and wide-field optical coherence tomography angiography (OCTA). Additionally, the patients’ history of hyperthyroidism, disease duration, thyroid function status, as well as external manifestations, such as strabismus and impaired ocular motility, were also documented. The patients with TED additionally underwent visual field tests and VEP measurements. Visual field testing was performed using automated perimetry (Humphrey Field Analyzer; Carl Zeiss Meditec Inc., Dublin, CA). The central 30-2 program was used with the standard Swedish Interactive Threshold Algorithm (SITA). Pattern visual evoked potential (P-VEP) recordings were measured according to the International Society for Clinical Electrophysiology of Vision (ISCEV) protocol.^9^ The potentials were elicited by checkerboard pattern reversal stimuli with large 1 degree (60 min arc) and small 0.25 degree (15 min arc) checks. P-VEPs used a repetition frequency at two reversals per second.

TED and DON were diagnosed according to European Group on Graves’ Orbitopathy (EUGOGO) criteria. Active TED was defined by a Clinical Activity Score (CAS) ≥3, whereas the diagnosis of DON was based on impaired visual function (reduced acuity, altered color vision, or visual field defects) and/or radiological evidence of apical crowding.^10^ CAS scores were assessed based on symptoms like eye pain, swelling, and redness. All patients with TED included in this study had not received corticosteroid therapy within 3 months prior to enrollment. Exclusion criteria included non-TED inflammatory orbital diseases, prior orbital decompression surgery, prior orbital radiotherapy, high intraocular pressure (>21 milimeters of mercury [mm Hg]), and ocular diseases affecting choroidal thickness (CT).

**Figure 1.**
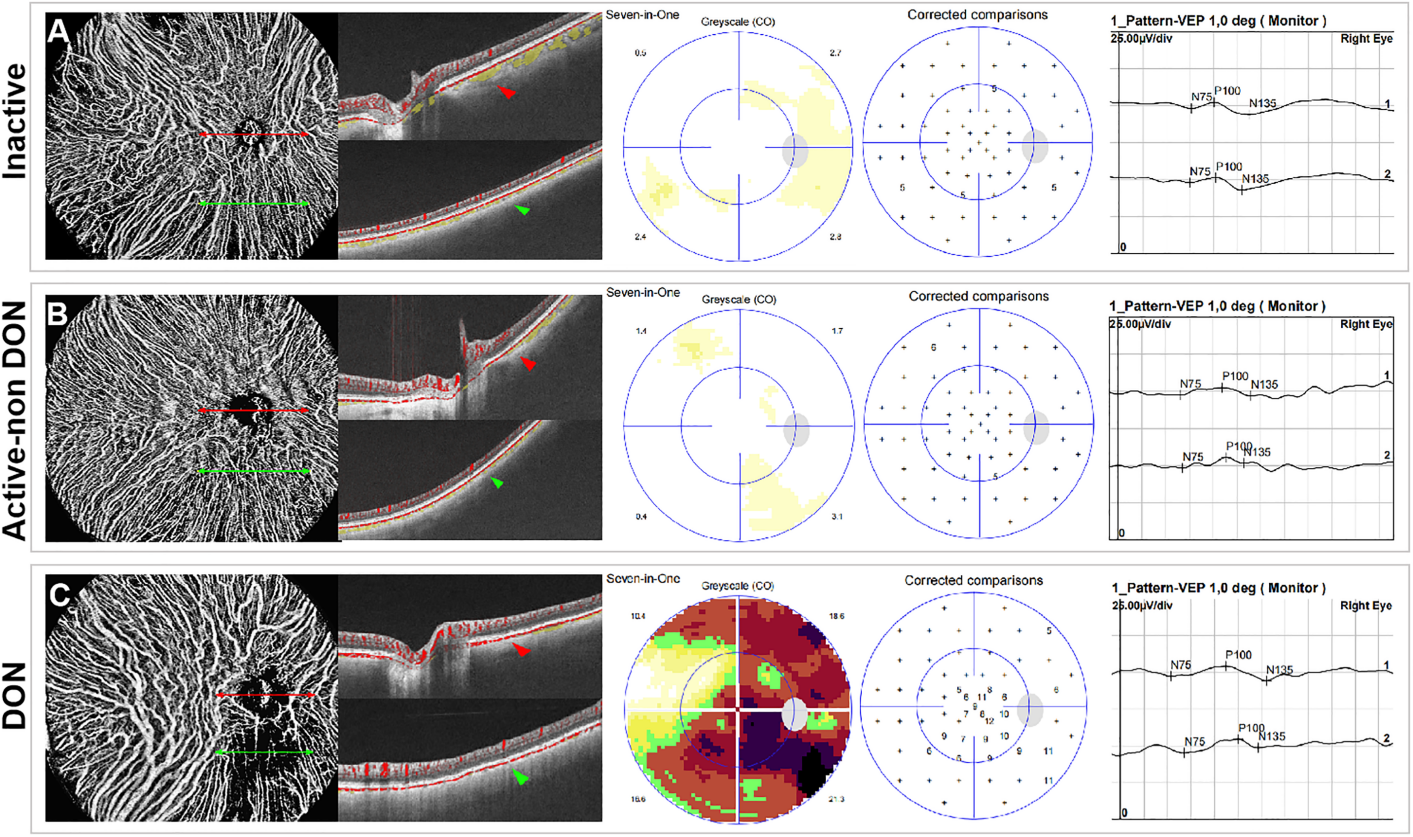
Representative choroidal and optic disc OCTA findings, visual field parameters, and VEP results in patients at different stages of TED. Panel **A** shows a patient with inactive TED, Panel **B** shows a patient with active TED without DON (active-nonDON), and Panel C shows a patient with DON. VEP, visual evoked potential; OCTA, optical coherence tomography angiography; TED, thyroid eye disease.

Choroidal and optic disc parameters were evaluated using 400-kHz swept-source (SS)-OCTA (TowardPi BMizar), with a 24 mm × 20 mm scanning range encompassing 1536 A-scans and 1280 B-scans. Initial segmentation was performed using the built-in automated software and subsequently reviewed for accuracy by an experienced ophthalmologist. The threshold for choroidal segmentation was dynamically determined, with adaptive adjustment of the choroidal signal threshold based on the signal intensity between the retinal pigment epithelium and Bruch's membrane. In cases of segmentation errors, trained technical personnel manually corrected the segmentation boundaries to ensure precise delineation of anatomic layers and enhance measurement reliability. Furthermore, all images were automatically graded by the system, and only those with a signal strength score greater than 9 (on a 10-point scale) were included in the final analysis. Thickness, volume, and blood flow density were derived from B-scans, offering a detailed view of the fundus (Fig. 1).

### Image Analysis

The three-dimensional choroidal data extraction in this study was carried out using measurements from the system’s built-in software. Following the methodology from previous research, the entire choroidal area was divided into a 3 × 3 grid, consisting of 9 regions: temporal-superior (TS), superior (S), nasal-superior (NS), temporal (T), macular (M), optic disc (OD), temporal-inferior (TI), inferior (I), and nasal-inferior (NI).^11^ The choroid is defined as the region from 29 µm below Bruch’s membrane to the choroid-sclera interface.

The system’s built-in software was used to measure choroidal and optic disc parameters. The choroidal parameters include CTchoroidal vascular volume (CVV), choroidal stromal volume (CSV), and choroidal vascular index (CVI). The optic disc parameters include optic disc area, cup volume, cup-to-disc area ratio, and blood flow density in the retinal nerve fiber layer (RNFL). The RNFL blood flow density refers to the proportion of the area exhibiting blood flow signals in the RNFL layer measured by optic disc OCTA, reflecting the blood flow perfusion status in the optic disc region.

### Statistical Analysis

Statistical analyses were performed using SPSS (version 26.0; IBM). Categorical data were presented as frequency counts, and continuous variables as mean ± standard deviation. For sample sizes greater than 50, normality was assessed using the Kolmogorov-Smirnov test; for samples smaller than 50, the Shapiro-Wilk test was used. ANOVA was used for normally distributed data, and the Kruskal-Wallis test for non-normally distributed data. Post hoc comparisons were done using Tukey's test. Pearson’s correlation coefficient assessed correlations between visual function and choroidal/optic disc parameters. To account for the potential correlation between fellow eyes, a sensitivity analysis was conducted. For this analysis, only the eye with more severe disease from each patient was included. This approach was designed to verify whether including both eyes in the primary analysis influenced the study results. A *P* value < 0.05 was considered significant.

### Ethics Approval Statements

All procedures were conducted in accordance with the principles of the Declaration of Helsinki. This study was approved by the Ethics Committee of Peking University Third Hospital (approval number: No. M2024461), and informed consent was obtained from all patients for the use of their clinical data.

### Patient and Public Involvement

Patients and the public were not involved in the design, conduct, reporting, or dissemination plans of this research.

## Results

### Clinical Characteristics of All Participants

A total of 38 patients with TED (76 eyes) were enrolled, with demographics in the Table. Groups included inactive (27 eyes), active non-DON (22 eyes), and DON (27 eyes). Figure 1 illustrates the changes in the choroid, visual field, and VEP across different stages of TED.

**Table.** Demographic Features of TED Subjects

	Inactive	Active Non-DON	DON	*P* Value
Eyes, *n*	27	22	27	/
Age, y	40.89 ± 11.66	46.05 ± 13.79	56.81 ± 8.10	<0.001***
Gender				
Male, *n*	6	5	15	/
Female, *n*	21	17	12	/
LogMAR VA	0.04 ± 0.06	0.13 ± 0.17	0.57 ± 0.42	<0.001***
SE, D	−3.75 ± −3.09	−1.26 ± −1.99	−1.31 ± −2.90	0.004**
Duration of TED, M	30.92 ± 17.93	16.25 ± 11.79	16.20 ± 11.53	0.001**
Corticosteroids				
YES, *n*	3	1	9	/
NO, *n*	24	21	16	/
Immunosuppressants				
YES, *n*	4	10	0	/
NO, *n*	23	12	27	/

Active non-DON, active thyroid eye disease without dysthyroid optic neuropathy group; D, diopters; DON, dysthyroid optic neuropathy group; Inactive, inactive thyroid eye disease group; *n*, number of eyes; SE, spherical equivalent; VA, visual acuity; y, year.

* *P* < 0.05, ** *P* < 0.01, *** *P* < 0.001.

### Comparison of BCVA, Pattern Visual Evoked Potential, and Visual Field Testing in Different Phases of TED

**Figure 2.**
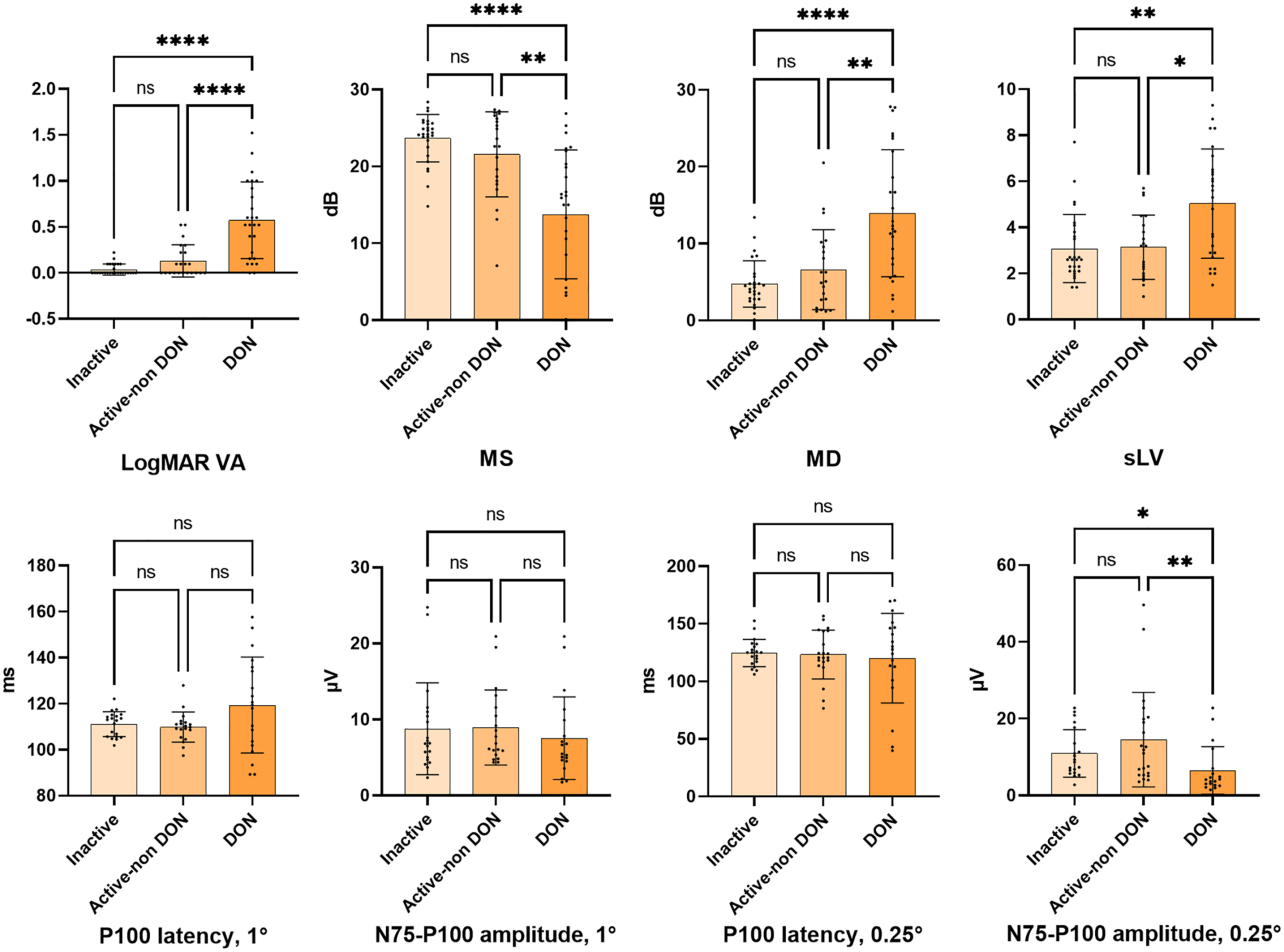
Differences in visual function parameters among groups at different stages of TED. VA, visual acuity; MS, mean sensitivity; MD, mean deviation; sLV, square root of loss variance; Inactive, inactive thyroid eye disease group; active non-DON, active thyroid eye disease without dysthyroid optic neuropathy group; DON, dysthyroid optic neuropathy group. *P < 0.05, **P < 0.01, ***P < 0.001, and ****P < 0.0001.

Figure 2 illustrates BCVA, P-VEP, and visual field parameters across TED stages. LogMAR visual acuity increased with TED severity (0.04 ± 0.06 vs. 0.13 ± 0.17 vs. 0.57 ± 0.42, *P* = 0.024). At 1.0 degrees, P-VEP showed no significant differences in P100 latency (*P* = 0.249) or N75-P100 amplitude (*P* = 0.196 to *P* = 0.693) across groups. At 0.25 degrees, the DON group exhibited significantly lower N75-P100 amplitudes (*P* < 0.001), averaging 3.66 ± 1.50 µV compared to 10.94 ± 6.20 µV (inactive) and 15.03 ± 12.35 µV (active non-DON). No significant differences in P100 latency were observed (*P* = 0.918).

In visual field tests, the DON group showed significantly lower mean sensitivity (14.31 ± 8.06 decibel [dB]) and higher mean defect (13.40 ± 7.93 dB) and squared loss variance (5.16 ± 2.34 dB) compared with inactive and active non-DON groups (*P* < 0.001 and *P* = 0.001, respectively). The reliability factor showed no significant differences (*P* = 0.529).

In the sensitivity analysis, including only the more severely affected eye per patient, the between-group differences in the primary outcomes remained statistically significant (*P* < 0.05). These findings indicate that the study conclusions were not affected by inter-eye correlation.

### Changes of Choroidal and Optic Disc Parameters in Different Phases of TED

Supplementary Table S3 summarizes changes in choroidal parameters and optic disc indices across TED stages. CVV decreased with increasing TED severity in the optic disc (3681.41 ± 1635.63, 3429.05 ± 1034.64, 2698.09 ± 1710.95 × 10⁶ µm³, *P* = 0.023), nasal-superior (3380.85 ± 1189.06, 3188.05 ± 952.99, 2607.96 ± 1531.76 × 10⁶ µm³, *P* = 0.024), and nasal-inferior regions (1662.59 ± 895.34, 2066.95 ± 830.50, 1490.74 ± 1053.85 × 10⁶ µm³, *P* = 0.028). Choroidal stroma volume also declined in the active non-DON and DON groups for wide-field (41,153.43 ± 9519.77 vs. 35,427.30 ± 11,009.30 × 10⁶ µm³, *P* = 0.032) and superior regions (5921.48 ± 1660.65 vs. 4874.91 ± 1795.05 × 10⁶ µm³, *P* = 0.032).

CVI decreased with TED severity in the macular (43.06 ± 3.54, 41.05 ± 2.78, 40.87 ± 2.97, *P* = 0.028), optic disc (43.95 ± 3.05, 41.60 ± 3.09, 40.95 ± 5.71, *P* = 0.015), and nasal-superior regions (45.65 ± 4.67, 42.52 ± 2.39, 41.46 ± 5.32, *P* = 0.003). Optic disc parameters showed no significant changes. ANCOVA with SE adjustment (Supplementary Table S4) revealed a decreasing trend in RNFL blood flow density across groups (47.05 ± 2.00, 46.67 ± 2.92, 45.29 ± 3.01, *P* = 0.038).

### Correlation Between Choroidal Parameters and Visual Function in TED

Supplementary Table S1 shows the correlation between choroidal blood flow parameters and logMAR visual acuity as well as P-VEP. In this regard, we found that among the choroidal parameters, CVV (*R* = −0.208, *P* = 0.082) and CSV (*R* = −0.272, *P* = 0.022) mainly showed a negative correlation with visual acuity. However, we did not observe a significant correlation between P-VEP and choroidal parameters.

**Figure 3.**
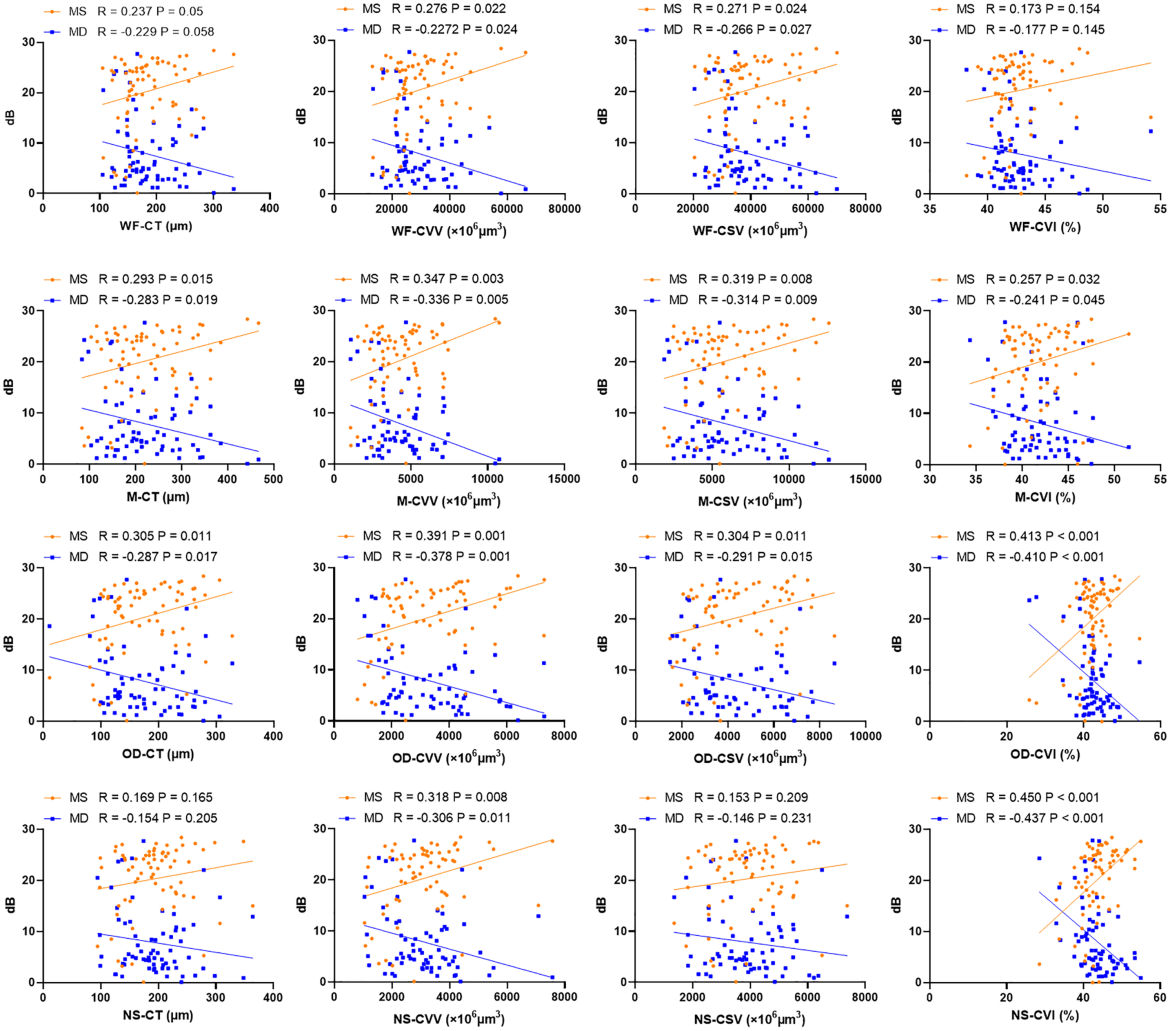
Correlation analysis between choroidal parameters and visual field parameters in patients with TED. CSV, choroidal stromal volume; CT, choroidal thickness; CVI, choroidal vascular index; CVV, choroidal vascular volume; MD, mean deviation; MS, mean sensitivity; NS, nasal-superior; OD, optic disc; WF, wide field.

Figure 3 primarily illustrates the correlation between visual field parameters and choroidal parameters. We found that CVV in both the wide-field (*R* = 0.276, *P* = 0.022), optic disc (*R* = 0.391, *P* = 0.001), and nasal superior (*R* = 0.318, *P* = 0.008) regions showed a positive correlation with mean sensitivity (MS). In addition, CSV in both the wide-field (*R* = 0.271, *P* = 0.024), and optic disc (*R* = 0.304, *P* = 0.011) regions also showed a positive correlation with MS. Moreover, we found the strongest correlation between CVI in the optic disc (*R* = 0.413, *P* < 0.001) and nasal superior (*R* = 0.450, *P* < 0.001) regions with MS, suggesting that CVI in these regions plays a significant role in predicting visual field function in patients.

### Correlation Between Optic Disc Parameters and Visual Function in TED

Supplementary Table S2 summarizes the correlation between optic disc and visual function parameters. A significant negative correlation was found between RNFL blood flow density and logMAR visual acuity (*R* = −0.372, *P* = 0.001), suggesting better visual acuity with higher RNFL blood flow.

Optic disc parameters showed varying correlations with P100 latency. Cup volume had a weak, near-significant negative correlation (*R* = −0.256, *P* = 0.054), as did the C/D area ratio (*R* = −0.260, *P* = 0.051). A significant negative correlation was observed for the horizontal C/D ratio (*R* = −0.264, *P* = 0.048), whereas the vertical C/D ratio showed a nonsignificant trend (*R* = −0.244, *P* = 0.068). Additionally, a weak but significant negative correlation was noted between optic disc area and N75-P100 amplitude during P-VEP at 15 minutes (*R* = −0.237, *P* = 0.048).

The OD area was negatively correlated with MS (*R* = −0.237, *P* = 0.048), but not with mean defect (MD; *R* = 0.225, *P* = 0.062). RNFL density showed a moderate positive correlation with N75-P100 amplitude at 0.25 degrees (*R* = 0.311, *P* = 0.021), and was significantly positively correlated with MS (*R* = 0.387, *P* = 0.001) and negatively with MD (*R* = −0.377, *P* = 0.001).

## Discussion

This study compared the efficacy of BCVA, visual field (VF), and VEP in distinguishing TED severity and examined their correlation with choroidal and optic disc parameters from wide-field OCTA. BCVA and VF worsened with increasing TED severity, whereas VEP had limited accuracy. BCVA and VF showed significant correlations with choroidal and optic disc parameters, whereas only the N75-P100 component of VEP was associated with RNFL blood flow density. This is the first study using wide-field OCTA to explore structural and vascular changes in TED and their relationship with visual function, highlighting the importance of early treatment to prevent permanent vision loss in DON. Thus, early and accurate diagnosis of DON is critical for initiating timely medical intervention.^4^ Although previous studies have suggested that no single visual test or optic nerve finding is sufficiently sensitive to diagnose or exclude DON, monitoring changes in visual function remains crucial in the management of TED.^3^^,^^12^^,^^13^ Our findings demonstrated a clear deterioration in both visual acuity and VF across groups as disease severity increased, consistent with previous studies.^14^^,^^15^ In contrast, VEP showed significant differences only in the N75-P100 amplitude, with variations primarily observed between the DON group and the other 2 groups. This indicates that VEP has limited value in differentiating inactive TED from active TED, as significant abnormalities are not apparent until the onset of DON. Previous studies have demonstrated that DON is characterized by decreased P100 amplitude and prolonged latency of both P100 and N75 compared with patients with TED or healthy controls.^3^^,^^16^^,^^17^ These studies did not differentiate between active and inactive TED stages. Our study adds to this by showing that, compared with visual acuity and VF, VEP exhibits greater variability, limiting its effectiveness as a sole indicator for tracking TED progression.

The observed associations between choroidal and optic disc alterations and visual function metrics suggest that CT and vascularity could serve as valuable biomarkers in the clinical assessment of DON. Incorporating wide-field OCTA measurements into routine evaluation may allow earlier detection of subclinical optic nerve compromise, better monitoring of disease progression, and more precise stratification of patients for targeted interventions. For example, patients exhibiting significant reductions in choroidal vascularity or blood flow density could be prioritized for closer follow-up or earlier therapeutic intervention. Furthermore, these findings highlight the potential for personalized management strategies, where choroidal metrics inform decisions regarding the timing and intensity of treatment, ultimately improving visual outcomes in patients with TED with or at risk of DON.

Previous studies on TED and OCT/OCTA have primarily concentrated on macular region changes, with limited exploration of alterations in broader ocular structures.^18^^–^^20^ Wide-field OCTA, as used in our study, provides a more comprehensive perspective on ocular structures, particularly emphasizing the vascular changes in the choroid and optic disc.^21^ Our findings revealed that CT, CSV, and CVV were decreased in the macular region, aligning with previous studies.^20^^,^^22^ These differences were not statistically significant. However, ANCOVA analysis in Supplementary Table S5 showed a decreasing trend in CVV and CVI in the macular region as TED severity increased. Wide-field OCTA revealed significant choroidal atrophy in the optic disc and nasal-superior region, indicating regional heterogeneity in choroidal deterioration as TED progresses. Previous studies have suggested that the thickening of the superior oblique muscle in the extraocular muscles is most closely associated with the development and progression of DON.^23^ This may explain why choroidal atrophy from compression is most pronounced in the nasal-superior and optic disc regions. Our study also suggests that choroidal parameters in these regions are crucial for detecting TED progression.

Additionally, an enlarged optic disc area and a reduction in RNFL blood flow density were observed. The consistent trends of increase or decrease suggested that structural and vascular changes in the optic disc play a crucial role in the progression of TED. Zhang et al. reported a significant reduction in optic nerve head vessel density in patients with DON compared with healthy controls and non-DON, which aligns with our findings.^24^ These findings collectively indicate that as TED progresses, the vessels of the optic nerve and the adjacent choroid exhibit consistent atrophic changes. Such changes may contribute to apical compression of the optic nerve, leading to subsequent optic nerve swelling or posterior ischemia.^7^^,^^25^ Although structural changes in the choroid and optic nerve demonstrate significant potential for predicting TED severity, further investigation is required to fully understand their relationship with alterations in visual function.

Previous studies on visual function tests and retinal OCTA parameters were limited to the macular or optic disc regions and lacked comprehensive multi-variable correlation analyses with various visual function metrics and OCT parameters.^24^^,^^26^^–^^28^ In our study, we were the first to investigate the relationship between wide-field choroidal OCTA parameters and visual function tests, including BCVA, VF, and VEP. Among these functional tests, VF demonstrated a broader and more significant correlation with choroidal indicators, highlighting its importance as a crucial monitoring tool closely associated with structural changes. Xu et al.^26^ reported similar findings, noting that VF-MD was significantly correlated with deep retinal and radial peripapillary capillary density, whereas BCVA and P100 latency showed no such correlation. However, Zhang et al.^24^ and Zeng et al.^29^ presented conflicting findings, reporting that P100 amplitude exhibited the highest correlation with macular vessel density. Notably, none of these studies specifically addressed choroidal changes, highlighting the need for further research to clarify the value of visual function tests in relation to structural changes.

Wide-field OCTA reveals strong associations between choroidal indicators in the optic disc and nasal-superior regions with VF and BCVA. RNFL blood flow density also correlated significantly with VF, BCVA, and N75-P100 amplitude in VEP tests. In previous studies, the blood supply to the optic disc has been closely correlated with the choroidal blood flow in the optic disc region and the blood flow in the RNFL layer.^30^ These findings reinforce the link between optic disc structural changes and visual function deterioration. Previous DON research highlighted optic disc congestion, edema, and atrophic changes in advanced stages.^31^ These findings highlight the need for regular assessment of both optic disc structure and function in monitoring TED progression. Additionally, the correlation between OCTA parameters and VF was stronger than with P-VEP, suggesting VF may play a more important role in evaluating visual function changes in TED.

Our findings offer insights into the relationship between OCT indicators and visual function in TED, but several limitations exist. The sample size may not fully represent TED's diverse stages, and the cross-sectional design limits causal inference. Future prospective studies with larger cohorts and longer follow-up are needed to validate these findings and explore OCTA's potential in monitoring TED progression. Moreover, the absence of a healthy control group limits our ability to determine the extent to which the observed choroidal alterations deviate from physiological conditions. Including age- and refractive error–matched healthy controls in future studies would help to better contextualize these findings. Furthermore, this was a single-center study conducted at a tertiary referral center, which may introduce selection bias toward more severe or atypical cases. Multicenter studies with more diverse patient populations are warranted to validate our results.

## Conclusions

This study integrated ultra-widefield OCTA-derived fundus structural parameters with visual function measures in patients with TED, demonstrating the concordance between visual impairment and choroidal ischemic atrophy caused by TED. The findings confirm that ultra-widefield OCTA parameters are valuable indicators of the severity of visual function damage in patients with TED and may serve as important monitoring metrics during future treatment.

## Supplementary Material

Supplement 1
