# Supplementary material for: Investigating the Correlation Between Choroidal Alteration and Visual Function Metrics in Dysthyroid Optic Neuropathy
Source: Transl Vis Sci Technol. 2026 Jun 29;15(6):38. doi: 10.1167/tvst.15.6.38 (PMC13326892; doi:10.1167/tvst.15.6.38)
Supplement: Supplement 1 [file tvst-15-6-38_s001.docx]

**Supplementary**

**Table S1 Correlation analysis between choroidal parameters and BCVA as well as P-VEP parameters.**

|  | | LogMAR VA | | P100 latency, 1° | | N75-P100 amplitude, 1° | | P100 latency, 0.25° | | N75-P100 amplitude, 0.25° | |
| --- | --- | --- | --- | --- | --- | --- | --- | --- | --- | --- | --- |
|  | | R | P | R | P | R | P | R | P | R | P |
| CT | Wide-Field | -0.189 | 0.114 | 0.117 | 0.382 | 0.011 | 0.935 | 0.015 | 0.91 | 0.026 | 0.852 |
|  | Macular | -0.227 | 0.057 | 0.07 | 0.599 | -0.006 | 0.965 | 0.016 | 0.902 | 0.008 | 0.954 |
|  | Optic disc | -0.21 | 0.078 | 0.17 | 0.202 | 0.001 | 0.993 | 0.079 | 0.551 | 0.119 | 0.383 |
|  | Nasal Superior | -0.124 | 0.303 | 0.223 | 0.093 | 0.076 | 0.571 | 0.02 | 0.878 | 0.101 | 0.458 |
| CVV | Wide-Field | -0.208 | 0.082 | 0.008 | 0.951 | 0.024 | 0.859 | -0.062 | 0.64 | 0.011 | 0.936 |
|  | Macular | -0.257 | 0.03* | -0.03 | 0.822 | 0.021 | 0.875 | -0.042 | 0.752 | 0.016 | 0.906 |
|  | Optic disc | -0.288 | 0.015* | 0.028 | 0.835 | 0.046 | 0.733 | -0.008 | 0.953 | 0.134 | 0.324 |
|  | Nasal Superior | -0.237 | 0.046* | 0.071 | 0.596 | 0.137 | 0.306 | -0.086 | 0.514 | 0.117 | 0.389 |
| CSV | Wide-Field | -0.272 | 0.022* | 0.017 | 0.902 | 0.076 | 0.573 | -0.028 | 0.834 | 0.099 | 0.47 |
|  | Macular | -0.31 | 0.009** | -0.019 | 0.89 | 0.053 | 0.691 | -0.042 | 0.751 | 0.069 | 0.613 |
|  | Optic disc | -0.276 | 0.02* | 0.061 | 0.651 | 0.051 | 0.701 | 0.016 | 0.904 | 0.178 | 0.188 |
|  | Nasal Superior | -0.213 | 0.075 | 0.055 | 0.681 | 0.155 | 0.246 | -0.04 | 0.763 | 0.189 | 0.162 |
| CVI | Wide-Field | 0.062 | 0.605 | -0.04 | 0.764 | -0.05 | 0.711 | -0.152 | 0.247 | -0.166 | 0.222 |
|  | Macular | 0.071 | 0.553 | -0.029 | 0.831 | -0.078 | 0.559 | 0.013 | 0.922 | -0.121 | 0.373 |
|  | Optic disc | -0.214 | 0.071 | -0.128 | 0.339 | 0.048 | 0.722 | -0.034 | 0.796 | -0.018 | 0.896 |
|  | Nasal Superior | -0.226 | 0.056 | 0.05 | 0.711 | 0.107 | 0.426 | -0.175 | 0.181 | -0.001 | 0.992 |

CT = Choroidal Thickness, CVV = Choroidal Vascular Volume, CSV = Choroidal Stromal Volume, CVI = Choroidal Vascular Index, WF = wide field, OD = optic disc, NS = nasal-superior. *P<0.05, **P<0.01, and ***P<0.001.

**Table S2 Correlation analysis between optic disc OCTA parameters and VEP as well as visual field parameters.**

|  | | Optic Disc Area | | Cup Volume | | Cup-to-disc area ratio | | Average RNFL blood flow density | |
| --- | --- | --- | --- | --- | --- | --- | --- | --- | --- |
|  |  | R | P | R | P | R | P | R | P |
| LogMAR VA | | 0.046 | 0.701 | -0.07 | 0.561 | -0.107 | 0.371 | -0.372 | **0.001**** |
| VEP 1° | P100 latency | 0.206 | 0.124 | -0.256 | 0.054 | -0.26 | 0.051 | -0.152 | 0.259 |
|  | N75-P100 amplitude | -0.226 | 0.091 | 0.009 | 0.945 | 0.166 | 0.218 | 0.18 | 0.181 |
| VEP 0.25° | P100 latency | -0.051 | 0.704 | -0.149 | 0.259 | -0.221 | 0.092 | 0.151 | 0.253 |
|  | N75-P100 amplitude | -0.237 | 0.081 | 0.04 | 0.775 | 0.132 | 0.338 | 0.311 | 0.021* |
| Octopus | MS | -0.237 | **0.048*** | 0.038 | 0.758 | 0.059 | 0.626 | 0.387 | **0.001**** |
|  | MD | 0.225 | 0.062 | -0.064 | 0.598 | -0.079 | 0.518 | -0.377 | **0.001**** |
|  | sLV | 0.209 | 0.083 | 0.026 | 0.829 | 0.017 | 0.891 | -0.151 | 0.213 |
|  | RF | 0.067 | 0.581 | -0.041 | 0.738 | -0.054 | 0.659 | -0.03 | 0.808 |

RNFL = retinal nerve fiber layer, MS = mean sensitivity, MD = mean deviation. **P*<0.05, ***P*<0.01, and ****P*<0.001.

**Table S3 Comparison of choroidal and optic disc parameters at different TED stages**

|  | | In-Active | Active-non DON | DON | *P* |
| --- | --- | --- | --- | --- | --- |
| CT (μm) | Wide-Field | 188.11 ± 54.07 | 189.67 ± 38.44 | 172.70 ± 51.02 | 0.108 |
|  | Macular | 236.22 ± 89.30 | 224.86 ± 71.61 | 208.30 ± 86.63 | 0.386 |
|  | Optic disc | 180.56 ± 62.05 | 183.57 ± 46.34 | 154.87 ± 78.14 | 0.124 |
|  | Nasal Superior | 197.15 ± 46.14 | 200.62 ± 49.64 | 175.70 ± 70.92 | 0.082 |
|  | Subfovea | 278.11 ± 119.14 | 251.71 ± 99.08 | 255.00 ± 93.29 | 0.629 |
|  | Tempo-superior | 210.89 ± 77.30 | 223.81 ± 57.18 | 211.96 ± 73.93 | 0.388 |
|  | Superior | 218.19 ± 60.67 | 225.33 ± 55.60 | 197.70 ± 68.20 | 0.079 |
|  | Tempo | 201.00 ± 66.60 | 193.86 ± 48.89 | 179.96 ± 48.40 | 0.31 |
|  | Tempo-inferior | 164.15 ± 53.14 | 163.10 ± 40.91 | 164.96 ± 41.45 | 0.991 |
|  | Inferior | 155.56 ± 54.01 | 149.48 ± 42.22 | 128.70 ± 51.68 | 0.149 |
|  | Nasal-inferior | 121.22 ± 37.76 | 139.48 ± 41.15 | 118.13 ± 52.05 | 0.11 |
| CVV  (×10^6^ μm^3^) | Wide-Field | 30821.48 ± 11871.72 | 30129.52 ± 7680.35 | 26701.83 ± 10023.23 | 0.082 |
|  | Macular | 4769.15 ± 2166.15 | 4292.76 ± 1621.19 | 3738.00 ± 1873.45 | 0.108 |
|  | Optic disc | 3681.41 ± 1635.63 | 3429.05 ± 1034.64 | 2698.09 ± 1710.95 | 0.023* |
|  | Nasal Superior | 3380.85 ± 1189.06 | 3188.05 ± 952.99 | 2607.96 ± 1531.76 | 0.024 |
|  | Tempo-superior | 3628.33 ± 1755.82 | 3774.62 ± 1217.72 | 4025.00 ± 2680.07 | 0.664 |
|  | Superior | 4441.74 ± 1573.11 | 4537.19 ± 1331.80 | 3917.17 ± 1744.67 | 0.061 |
|  | Tempo | 3737.04 ± 1812.49 | 3568.00 ± 1156.65 | 3265.87 ± 1177.72 | 0.429 |
|  | Tempo-inferior | 2551.59 ± 1094.76 | 2499.81 ± 936.29 | 2563.91 ± 838.23 | 0.973 |
|  | Inferior | 2968.78 ± 1473.65 | 2773.10 ± 1010.85 | 2395.09 ± 1115.57 | 0.213 |
|  | Nasal-inferior | 1662.59 ± 895.34 | 2066.95 ± 830.50 | 1490.74 ± 1053.85 | 0.028* |
| CSV  (×10^6^ μm^3^) | Wide-Field | 39766.96 ± 12420.78 | 41153.43 ± 9519.77 | 35427.30 ± 11009.30 | 0.032* |
|  | Macular | 6269.85 ± 2676.79 | 6143.05 ± 2242.20 | 5381.83 ± 2642.55 | 0.275 |
|  | Optic disc | 4573.22 ± 1681.83 | 4804.33 ± 1479.85 | 3775.09 ± 2069.21 | 0.051 |
|  | Nasal Superior | 3937.85 ± 906.82 | 4290.38 ± 1240.52 | 3499.74 ± 1525.79 | 0.052 |
|  | Tempo-superior | 4250.26 ± 1664.89 | 4649.67 ± 1310.35 | 4486.22 ± 1659.37 | 0.236 |
|  | Superior | 5632.00 ± 1739.07 | 5921.48 ± 1660.65 | 4874.91 ± 1795.05 | 0.037* |
|  | Tempo | 5272.70 ± 1925.70 | 5211.76 ± 1484.06 | 4740.70 ± 1365.42 | 0.369 |
|  | Tempo-inferior | 3448.04 ± 1373.14 | 3564.29 ± 946.08 | 3379.13 ± 1007.06 | 0.525 |
|  | Inferior | 3753.59 ± 1430.11 | 3617.33 ± 1270.47 | 2985.48 ± 1133.69 | 0.086 |
|  | Nasal-inferior | 2629.44 ± 938.16 | 2951.14 ± 1068.26 | 2304.22 ± 1167.84 | 0.072 |
| CVI  (%) | Wide-Field | 43.23 ± 2.42 | 42.10 ± 1.46 | 42.59 ± 3.26 | 0.155 |
|  | Macular | 43.06 ± 3.54 | 41.05 ± 2.78 | 40.87 ± 2.97 | 0.028* |
|  | Optic disc | 43.95 ± 3.05 | 41.60 ± 3.09 | 40.95 ± 5.71 | 0.015* |
|  | Nasal Superior | 45.65 ± 4.67 | 42.52 ± 2.39 | 41.46 ± 5.32 | 0.003** |
|  | Tempo-superior | 45.12 ± 4.84 | 44.55 ± 2.91 | 44.92 ± 8.12 | 0.231 |
|  | Superior | 43.88 ± 2.95 | 43.25 ± 1.99 | 44.15 ± 3.42 | 0.573 |
|  | Tempo | 40.62 ± 4.11 | 40.17 ± 3.23 | 40.46 ± 4.40 | 0.925 |
|  | Tempo-inferior | 42.07 ± 3.15 | 40.30 ± 4.30 | 42.88 ± 4.70 | 0.106 |
|  | Inferior | 42.95 ± 4.91 | 43.38 ± 2.46 | 43.71 ± 4.21 | 0.806 |
|  | Nasal-inferior | 37.21 ± 5.92 | 40.77 ± 2.67 | 37.29 ± 4.98 | 0.025* |
| optic disc | Optic disc area (mm^2^) | 1.53 ± 0.52 | 1.68 ± 0.55 | 1.95 ± 1.20 | 0.064 |
|  | Cup volume (mm^3^) | 0.10 ± 0.13 | 0.15 ± 0.17 | 0.11 ± 0.12 | 0.747 |
|  | Cup-to-disc area ratio | 0.33 ± 0.22 | 0.43 ± 0.32 | 0.37 ± 0.22 | 0.657 |
|  | Average RNFL blood flow density (%) | 47.05 ± 2.00 | 46.67 ± 2.92 | 45.29 ± 3.01 | 0.075 |

CT = choroid thickness, CVV = choroidal vascular volume, CSV = choroidal stromal volume, CVI = choroidal vascular index, Inactive = Inactive thyroid eye disease group, active-non DON = active thyroid eye disease without dysthyroid optic neuropathy group, DON = dysthyroid optic neuropathy group. **P*<0.05, ***P*<0.01, and ****P*<0.001.

**Table S4 ANCOVA based on SE adjustment for choroidal and optic disc parameters at different stages of TED**

|  | | In-Active | Active-non DON | DON | **ANCOVA**  *P* |
| --- | --- | --- | --- | --- | --- |
| CT | Wide-Field | 188.11 ± 54.07 | 189.67 ± 38.44 | 172.70 ± 51.02 | 0.054 |
|  | Macular | 236.22 ± 89.30 | 224.86 ± 71.61 | 208.30 ± 86.63 | 0.085 |
|  | Optic disc | 180.56 ± 62.05 | 183.57 ± 46.34 | 154.87 ± 78.14 | 0.125 |
|  | Nasal Superior | 197.15 ± 46.14 | 200.62 ± 49.64 | 175.70 ± 70.92 | 0.085 |
|  | Subfovea | 278.11 ± 119.14 | 251.71 ± 99.08 | 255.00 ± 93.29 | 0.041* |
|  | Tempo-superior | 210.89 ± 77.30 | 223.81 ± 57.18 | 211.96 ± 73.93 | 0.358 |
|  | Superior | 218.19 ± 60.67 | 225.33 ± 55.60 | 197.70 ± 68.20 | 0.072 |
|  | Tempo | 201.00 ± 66.60 | 193.86 ± 48.89 | 179.96 ± 48.40 | 0.096 |
|  | Tempo-inferior | 164.15 ± 53.14 | 163.10 ± 40.91 | 164.96 ± 41.45 | 0.421 |
|  | Inferior | 155.56 ± 54.01 | 149.48 ± 42.22 | 128.70 ± 51.68 | 0.004** |
|  | Nasal-inferior | 121.22 ± 37.76 | 139.48 ± 41.15 | 118.13 ± 52.05 | 0.286 |
| CVV | Wide-Field | 30821.48 ± 11871.72 | 30129.52 ± 7680.35 | 26701.83 ± 10023.23 | 0.021* |
|  | Macular | 4769.15 ± 2166.15 | 4292.76 ± 1621.19 | 3738.00 ± 1873.45 | 0.021* |
|  | Optic disc | 3681.41 ± 1635.63 | 3429.05 ± 1034.64 | 2698.09 ± 1710.95 | 0.017* |
|  | Nasal Superior | 3380.85 ± 1189.06 | 3188.05 ± 952.99 | 2607.96 ± 1531.76 | 0.017* |
|  | Tempo-superior | 3628.33 ± 1755.82 | 3774.62 ± 1217.72 | 4025.00 ± 2680.07 | 0.596 |
|  | Superior | 4441.74 ± 1573.11 | 4537.19 ± 1331.80 | 3917.17 ± 1744.67 | 0.059 |
|  | Tempo | 3737.04 ± 1812.49 | 3568.00 ± 1156.65 | 3265.87 ± 1177.72 | 0.104 |
|  | Tempo-inferior | 2551.59 ± 1094.76 | 2499.81 ± 936.29 | 2563.91 ± 838.23 | 0.341 |
|  | Inferior | 2968.78 ± 1473.65 | 2773.10 ± 1010.85 | 2395.09 ± 1115.57 | 0.003** |
|  | Nasal-inferior | 1662.59 ± 895.34 | 2066.95 ± 830.50 | 1490.74 ± 1053.85 | 0.130 |
| CSV | Wide-Field | 39766.96 ± 12420.78 | 41153.43 ± 9519.77 | 35427.30 ± 11009.30 | 0.030* |
|  | Macular | 6269.85 ± 2676.79 | 6143.05 ± 2242.20 | 5381.83 ± 2642.55 | 0.094 |
|  | Optic disc | 4573.22 ± 1681.83 | 4804.33 ± 1479.85 | 3775.09 ± 2069.21 | 0.079 |
|  | Nasal Superior | 3937.85 ± 906.82 | 4290.38 ± 1240.52 | 3499.74 ± 1525.79 | 0.063 |
|  | Tempo-superior | 4250.26 ± 1664.89 | 4649.67 ± 1310.35 | 4486.22 ± 1659.37 | 0.587 |
|  | Superior | 5632.00 ± 1739.07 | 5921.48 ± 1660.65 | 4874.91 ± 1795.05 | 0.033* |
|  | Tempo | 5272.70 ± 1925.70 | 5211.76 ± 1484.06 | 4740.70 ± 1365.42 | 0.135 |
|  | Tempo-inferior | 3448.04 ± 1373.14 | 3564.29 ± 946.08 | 3379.13 ± 1007.06 | 0.437 |
|  | Inferior | 3753.59 ± 1430.11 | 3617.33 ± 1270.47 | 2985.48 ± 1133.69 | 0.002** |
|  | Nasal-inferior | 2629.44 ± 938.16 | 2951.14 ± 1068.26 | 2304.22 ± 1167.84 | 0.116 |
| CVI | Wide-Field | 43.23 ± 2.42 | 42.10 ± 1.46 | 42.59 ± 3.26 | 0.027* |
|  | Macular | 43.06 ± 3.54 | 41.05 ± 2.78 | 40.87 ± 2.97 | 0.040* |
|  | Optic disc | 43.95 ± 3.05 | 41.60 ± 3.09 | 40.95 ± 5.71 | <0.001*** |
|  | Nasal Superior | 45.65 ± 4.67 | 42.52 ± 2.39 | 41.46 ± 5.32 | 0.001** |
|  | Tempo-superior | 45.12 ± 4.84 | 44.55 ± 2.91 | 44.92 ± 8.12 | 0.778 |
|  | Superior | 43.88 ± 2.95 | 43.25 ± 1.99 | 44.15 ± 3.42 | 0.225 |
|  | Tempo | 40.62 ± 4.11 | 40.17 ± 3.23 | 40.46 ± 4.40 | 0.618 |
|  | Tempo-inferior | 42.07 ± 3.15 | 40.30 ± 4.30 | 42.88 ± 4.70 | 0.054 |
|  | Inferior | 42.95 ± 4.91 | 43.38 ± 2.46 | 43.71 ± 4.21 | 0.687 |
|  | Nasal-inferior | 37.21 ± 5.92 | 40.77 ± 2.67 | 37.29 ± 4.98 | 0.060 |
| Optic Disc  OCTA | Optic disc area (mm2) | 1.53 ± 0.52 | 1.68 ± 0.55 | 1.95 ± 1.20 | 0.394 |
|  |  |  |  |  |  |
|  | Cup volume (mm3) | 0.10 ± 0.13 | 0.15 ± 0.17 | 0.11 ± 0.12 | 0.613 |
|  | Cup-to-disc area ratio | 0.33 ± 0.22 | 0.43 ± 0.32 | 0.37 ± 0.22 | 0.393 |
|  | Average RNFL blood flow density (%) | 47.05 ± 2.00 | 46.67 ± 2.92 | 45.29 ± 3.01 | 0.038* |

Inactive = Inactive thyroid eye disease group, active-non DON = active thyroid eye disease without dysthyroid optic neuropathy group, DON = dysthyroid optic neuropathy group, CVV = choroidal vascular volume, CSV = choroidal stromal volume, CVI = choroidal vascular index. **P*<0.05, ***P*<0.01, and ****P*<0.001.
